# Cobalamin *cbiP* mutant shows decreased tolerance to low temperature and copper stress in *Listeria monocytogenes*

**DOI:** 10.1186/s40659-022-00376-4

**Published:** 2022-03-02

**Authors:** L. Vásquez, A. Parra, A. M. Quesille-Villalobos, G. Gálvez, P. Navarrete, M. Latorre, M. Toro, M. González, A. Reyes-Jara

**Affiliations:** 1 Laboratorio de Microbiología y Probióticos, INTA Universidad de Chile, Avenida El Líbano 5524 Macul, Santiago, Chile; 2 Doctorado en Acuicultura, Programa Cooperativo Universidad de Chile, Universidad Católica del Norte, Pontificia Universidad Católica de Valparaíso, Valparaíso, Chile; 3 Facultad de Ciencias del Mar, Universidad Católica del Norte, Larrondo 1281, Coquimbo, Chile; 4 Laboratorio de Bioingeniería, Instituto de Ciencias de la Ingeniería, Universidad de O’Higgins, Rancagua, Chile; 5 ANID-Millennium Science Initiative Program—Millennium Nucleus in the Biology of the Intestinal Microbiota, Santiago, Chile; 6 Laboratorio de Bioinformática y Expresión Génica, INTA, Universidad de Chile, Santiago, Chile; 7 Fondap Center for Genome Regulation (CGR), Santiago, Chile

**Keywords:** *Listeria monocytogenes*, Copper, Cobalamin, Low temperature

## Abstract

**Background:**

*Listeria monocytogenes* is a foodborne pathogen that causes listeriosis in humans. This pathogen activates multiple regulatory mechanisms in response to stress, and cobalamin biosynthesis might have a potential role in bacterial protection. Low temperature is a strategy used in the food industry to control bacteria proliferation; however, *L. monocytogenes* can grow in cold temperatures and overcome different stress conditions. In this study we selected *L. monocytogenes* List2-2, a strain with high tolerance to the combination of low temperature + copper, to understand whether the cobalamin biosynthesis pathway is part of the tolerance mechanism to this stress condition. For this, we characterized the transcription level of three cobalamin biosynthesis-related genes (*cbiP*, *cbiB,* and *cysG*) and the *eutV* gene, a transcriptional regulator encoding gene involved in ethanolamine metabolism, in *L. monocytogenes* strain List2-2 growing simultaneously under two environmental stressors: low temperature (8 °C) + copper (0.5 mM of CuSO_4_ × 5H_2_O). In addition, the gene *cbiP*, which encodes an essential cobyric acid synthase required in the cobalamin pathway, was deleted by homologous recombination to evaluate the impact of this gene in *L. monocytogenes* tolerance to a low temperature (8 °C) + different copper concentrations.

**Results:**

By analyzing the KEGG pathway database, twenty-two genes were involved in the cobalamin biosynthesis pathway in *L. monocytogenes* List2-2. The expression of genes *cbiP*, *cbiB,* and *cysG,* and *eutV* increased 6 h after the exposure to low temperature + copper. The cobalamin *cbiP* mutant strain List2-2Δ*cbiP* showed less tolerance to low temperature + copper (3 mM) than the wild-type *L. monocytogenes* List2-2. The addition of cyanocobalamin (5 nM) to the medium reverted the phenotype observed in List2-2Δ*cbiP*.

**Conclusion:**

These results indicate that cobalamin biosynthesis is necessary for *L. monocytogenes* growth under stress and that the *cbiP* gene may play a role in the survival and growth of *L. monocytogenes* List2-2 at low temperature + copper.

**Supplementary Information:**

The online version contains supplementary material available at 10.1186/s40659-022-00376-4.

## Background

Foodborne diseases are a significant cause of morbidity and mortality worldwide, and they are considered a major public health problem [1–3]. One of the most relevant foodborne pathogens is *Listeria monocytogenes,* a ubiquitous microorganism [4–6]. *L. monocytogenes* causes listeriosis in humans, a disease acquired through the ingestion of contaminated food. Listeriosis can range from febrile gastroenteritis to severe invasive disease; it primarily affects neonates, the immunosuppressed, pregnant women, and the elderly, reaching mortality rates up to 30% [5, 7].

*Listeria monocytogenes* can survive and grow under diverse stress conditions; it can tolerate and adapt to high NaCl concentrations (≥ 10%), a wide range of pH (4.5–9.0), and several sanitizers [4, 8, 9]. *L. monocytogenes* can grow at temperatures as low as − 1 °C [8]. The pathogen has been isolated from soil, silage, seawater, estuarine water, surface waters, and most importantly, food processing facilities and diverse food matrices [10–14]. The genetic characteristics of *L. monocytogenes* and the regulation of the transcriptional response are the main reasons why this bacterium survives in a wide range of environmental conditions [5, 15, 16].

Cobalamin–vitamin B12—is a water-soluble vitamin synthesized by bacteria and archaea [17]. As a nutrient, cobalamin acts as a cofactor for methyltransferases, isomerases, and dehalogenases, all enzymes involved in several essential biochemical processes such as carbon source fermentation and ribonucleotide reduction [18, 19]. Cobalamin and its derivatives are involved in the fermentation of small molecules such as 1,2-propanediol and ethanolamine [20]. Cobalamin biosynthesis is a complex process; more than thirty genes have been linked to its biosynthesis. Reports indicate that only a few bacteria and archaea can synthesize this molecule [21, 22]. De novo biosynthesis occurs as an anaerobic process in archaea and aerobic/anaerobic in bacteria [23, 24].

Ferrer et al. described that cobalamin acts as an antioxidant agent in *Leptospirillum* [25]. This iron-oxidizing bacterium belongs to the phylum *Nitrospirae* and grows in highly acidic and metal-loaded environments. When *Leptospirillum* was exposed for 1 h to oxidative stress conditions (Fe_2_(SO_4_)_3_, H_2_O_2_ or K_2_CrO_4_), intracellular reactive oxygen species (ROS) increased significantly; however, when the bacterium’s growth medium was supplemented with cobalamin before the stress, ROS levels remained at basal level [25]. *L. monocytogenes* can also grow under different stress conditions, such as the acidic pH used as a strategy to control bacterial growth along the food chain [14]. It has been described that cobalamin, 1,2-propanediol, and ethanolamine may help *L. monocytogenes* survive in food and food production environments [26, 27]. A global transcriptional study of *L. monocytogenes* growing in vacuum-packed salmon at 7 °C *versus* modified brain–heart infusion broth at 7 °C revealed that 149 genes changed their expression. Among the up-regulated genes (n = 88), twenty-six encoded proteins related to cobalamin biosynthesis, ethanolamine metabolism, and 1,2-propanediol [28].

New alternatives to control *L. monocytogenes* in the food industry have been explored. One strategy is using copper, which has antibacterial activity on different pathogenic bacteria [29–31]. Although copper has been described as an essential micronutrient that participates as an enzyme cofactor [32, 33], its intracellular level is strictly regulated because its excess is toxic [34, 35]. We previously observed that the antibacterial effect of copper in *L. monocytogenes* was enhanced at low temperatures [35] and we determined that the minimal inhibitory concentration of copper at 37 °C was 10–12 mM and at 8 °C was 4–6 mM of CuSO_4_ × 5H_2_O [36].

We analyzed the proliferation rate of different strains of *L. monocytogenes* and identified that the List2-2 strain had the highest growth rate at 8 °C [37]. This strain was the least affected by the combination of low temperature + copper according to their growth kinetic parameters [36]. List2-2 was selected to evaluate the effect of the global transcriptional response to low temperature and copper. This strain modified the expression of 263 genes in response to a sub-inhibitory copper concentration (0.5 mM of CuSO_4_ × 5H_2_O) + low temperature (8 °C), including some genes encoding for proteins involved in the cobalamin biosynthesis pathway [36]. Based on this information, we hypothesized that cobalamin biosynthesis was involved in the tolerance of *L. monocytogenes* List2-2 to the simultaneous exposure to low temperature (8 °C) + copper (0.5 mM) as a stress condition. In this study, we identified genes involved in cobalamin biosynthesis by analyzing the KEGG database and quantified the abundance of transcripts for genes associated with this pathway at 8 °C + copper over time. Finally, we mutated *cbiP,* which encodes for a cobyric acid synthase involved in the cobalamin biosynthesis, and studied the mutant’s phenotype.

## Results

### Genes involved in the cobalamin biosynthesis in *L. monocytogenes* List2-2

All the elements described for cobalamin biosynthesis in *L. monocytogenes* EGD-e were identified in List2-2 (Additional file 1: Fig. S1), and we identified 22 cobalamin biosynthesis genes. All 22 genes showed 100% identity with those described in the *L. monocytogenes* EGD-e strain in the KEGG database. Two upstream genes of the cobalamin biosynthetic pathway were located in the same strand as an operon responsible for the metabolism of 1,2-propanediol. The transcriptional regulator encoding gene *eutV* was also identified; this gene is responsible for the transcription of the ethanolamine operon and is regulated by cobalamin.

### Expression pattern of cobalamin-related genes in *L. monocytogenes* under simultaneous exposure to low temperature + copper

**Fig. 1:**
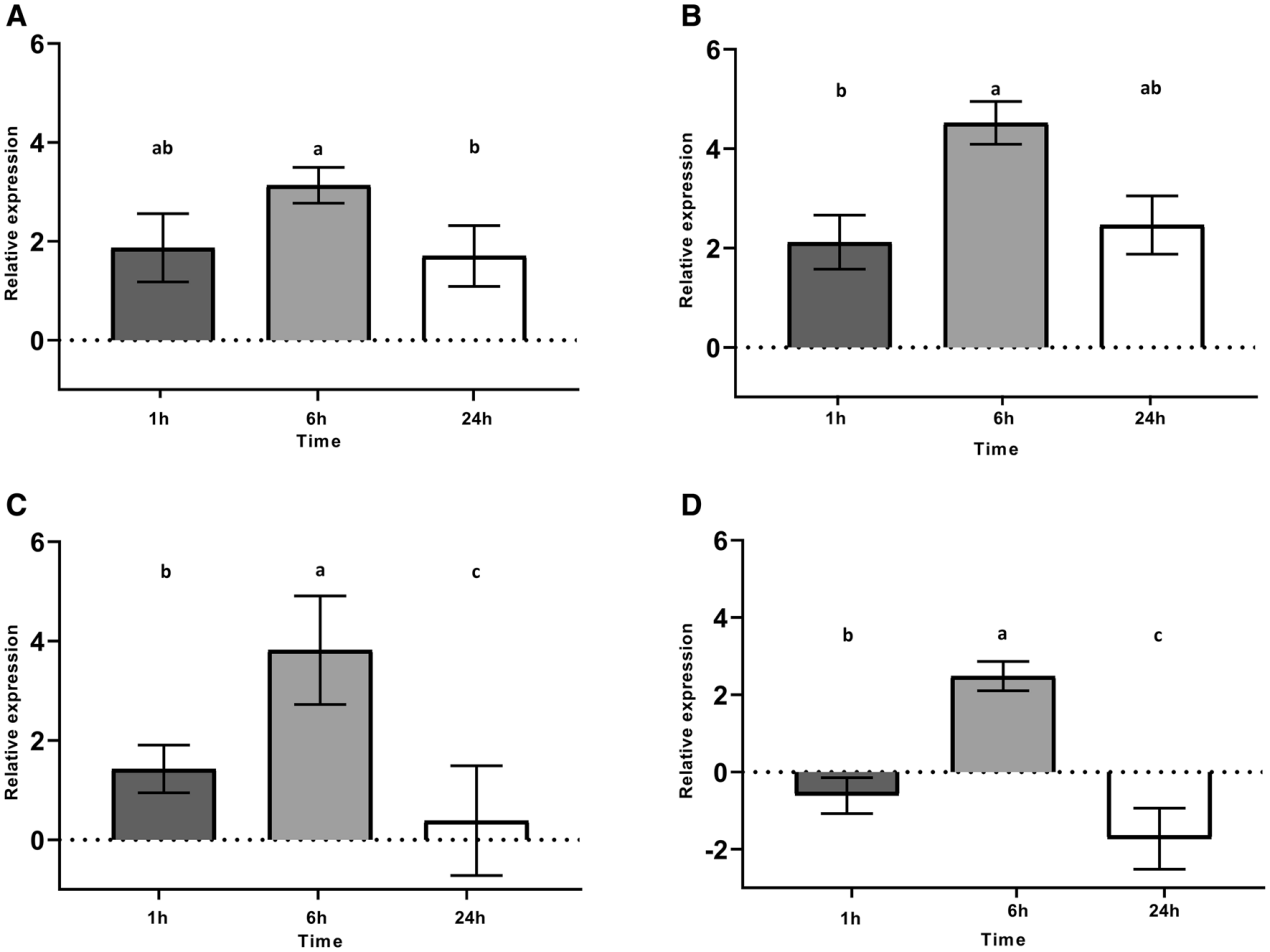
Temporal expression pattern of cobalamin biosynthesis-related genes in response to copper (0.5 mM) of *L. monocytogenes* List2-2 growing at 8 °C. **A** *cysG*, **B** *cbiP*, **C** *cbiB*, **D** *eutV*. Data were expressed as fold-change between copper treated at different times and untreated (time zero without copper). Different letters indicate significant differences comparing different times (1, 6 and 24 h). *P-value*: < 0.05

We selected three genes involved in cobalamin biosynthesis to evaluate their transcriptional response to low temperature (8 °C) + copper (0.5 mM) simultaneously at different times of exposure (Fig. 1). The genes *lmo1201* (*cysG)*, *lmo1208* (*cbiP*) and *lmo1192* (*cbiB*) were chosen based on their genome location in the cobalamin biosynthesis gene cluster and for being involved in the first and last stages of cobalamin biosynthesis (Additional file 1: Fig. S1). We observed that the relative expression of the genes did not change after 1 h of simultaneous exposure at 8 °C + copper (0.5 mM) compared to the control (8 °C, no copper). A significant increase in the relative expression was observed for the genes *cysG*, *cbiP,* and *cbiB* at 6 h after copper exposure*,* but this expression decreased after 24 h of exposure at the level of 1 h. Interestingly, *cbiP* presented 4.5 times the abundance of transcripts of the control at 6 h. The gene *eutV*, a transcriptional regulator of ethanolamine metabolism, presented the same expression pattern observed for *cbiP*, with a significant increase at 6 h of exposure and a reduction at 24 h (Fig. 1D).

### *L. monocytogenes* List2-2Δ*cbiP* showed lower tolerance to low temperature + copper

**Fig. 2:**
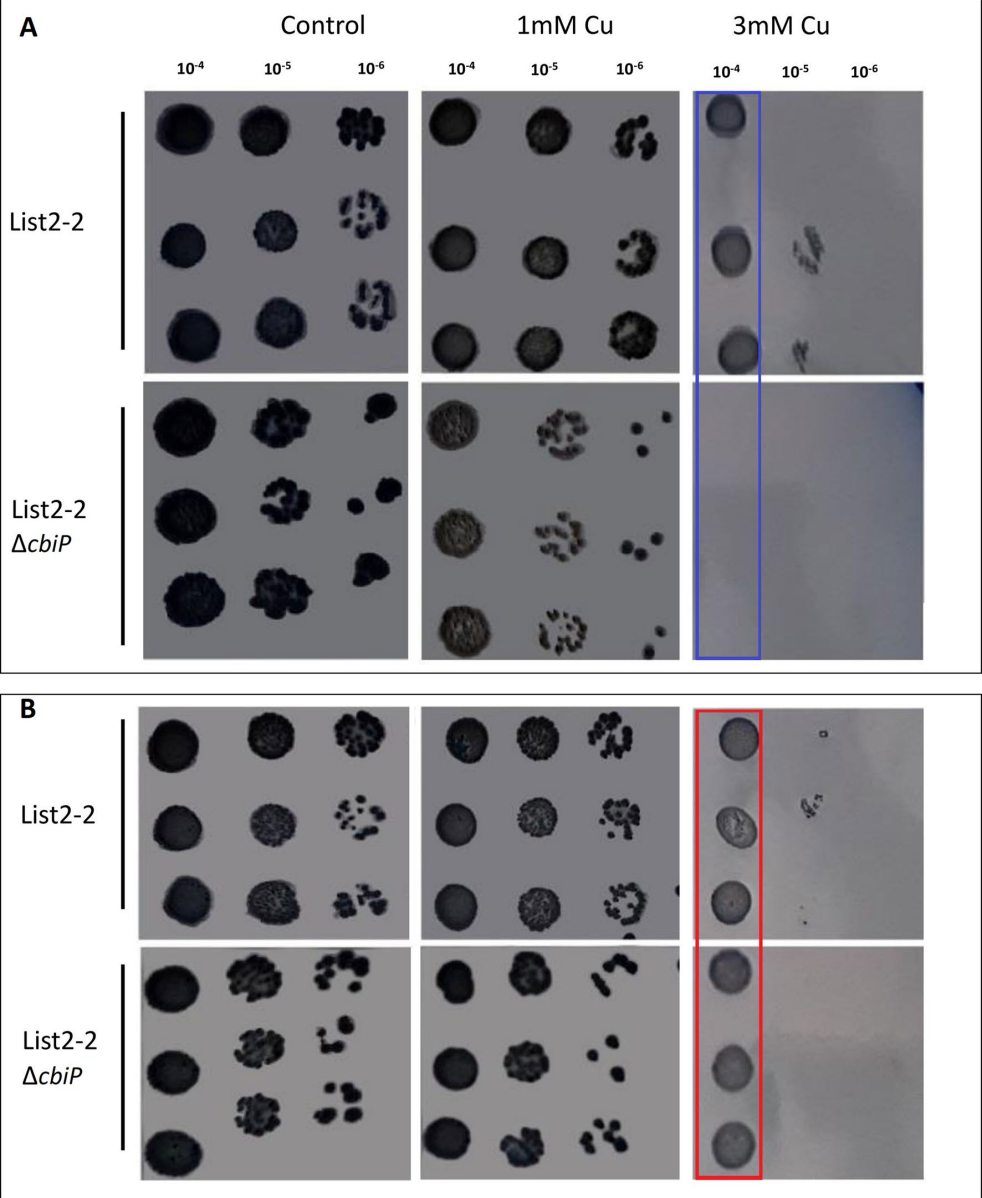
Effect of different copper concentrations on the growth of *L. monocytogenes* strain List2-2 and List2-2Δ*cbiP* cultured in TSBYe agar at 8 °C without (**A**) or with (**B**) cyanocobalamin 5 nM cyanocobalamin. The blue rectangle shows the differences in bacterial growth between List2-2 wild-type and List2-2Δ*cbiP* in agar media without cobalamin. The red rectangle shows the bacterial growth between List2-2 wild-type and List2-2Δ*cbiP* in agar media with cobalamin

To evaluate the importance of genes encoding for cobalamin biosynthesis in copper tolerance at low temperature, we deleted the gene *cbiP* by homologous recombination. As a result, List2-2Δ*cbiP* did not grow in agar media supplemented with 3 mM of copper at 8 °C, while the wild-type strain proliferated under the same condition (Fig. 2A). This result suggests that List2-2Δ*cbiP* is more sensitive to copper at low temperature than the wild-type strain on a solid medium. To evaluate the effect of cobalamin on the mutant, we supplemented the medium with cyanocobalamin (the active form of cobalamin). In this condition, List2-2Δ*cbiP* was able to grow at 3 mM copper cultured at 8 °C in the solid medium, demonstrating that 5 nM of cyanocobalamin improved List2-2Δ*cbiP* survival cultured at low temperature + copper (Fig. 2B).

## Discussion

One of the mechanisms that microorganisms use to respond to external stimuli is by modifying the expression of genes that encode proteins that allow them to adapt to new environments. The ability of *L. monocytogenes* to multiply at low temperatures and tolerate different stress factors is a consequence of the diversity of genetic elements that it encodes in its genome and the regulation that controls the expression of these elements. Among these components are elements that code for cobalamin synthesis, a cofactor that participates in different metabolic processes and plays a fundamental role in the pathogen's survival under stress conditions [38].

Cobalamin is synthesized by certain bacteria and archaea, but not by plants or animals; in higher organisms, the requirements of this vitamin are covered by interactions between microorganisms, plants, and animal tissues that are part of the food chain [38, 39]. It has been proposed that the evolution of the cyanocobalamin synthesis pathway allowed the fermentation of small molecules such as ethanolamine, 1,2-propanediol, and glycerol in anaerobic environments [20]. De novo cobalamin synthesis occurs in bacteria and archaea, and some species also may synthesize cobalamin by absorbing corrinoids through the salvage pathway [40].

The genes involved in cobalamin biosynthesis in *L. monocytogenes* are conserved [26]. The genome sequence analysis of List 2-2 strain allowed us to identify the 22 genes previously described involved in the anaerobic pathway of cobalamin biosynthesis. We have observed that the growth of *L. monocytogenes* in low temperature and copper activates mechanisms in response to stress that allow the bacterium to tolerate these conditions [36, 37]. Recently, Ahn et al*.* [41] observed that cobalamin biosynthesis acted as a protective mechanism against the oxidative stress generated by cold in *Thioalkalivibrio* spp. (haloalkaliphilic chemolithoautotrophic sulfur-oxidizing) growing at low temperature (10 °C). It has been reported that cobalamin biosynthesis genes increase their expression in response to arsenic, highlighting the antioxidant activity of this vitamin against metal stress [42].

Information about the relationship between cobalamin and the stress response in *L. monocytogenes* is limited. Studies reporting *L. monocytogenes* exposed to quaternary ammonium compounds reported a significant increase in expression for cobalamin genes [43]. Similar effects were detected when *L. monocytogenes* was cold-stressed under vacuum conditions [28]. Recently, transcriptomics of *L. monocytogenes* co-cultured with cheese rind bacteria revealed that genes of the ethanolamine, 1,2-propanediol, and cobalamin metabolism were up-regulated, showing that these genes were fundamental in a competitive environment against other bacteria [27].

Previously we had observed that the transcriptional response of *L. monocytogenes* differed when stress factors were presented separately or together. In particular, exposure of List2-2 to low temperature + copper activated different cellular processes from those regulated by cold or copper [36]. In this study, we observed an increase in the expression of genes associated with the cobalamin pathway when *L. monocytogenes* was cultured at different times at low temperatures with copper in aerobic conditions. This observation could indicate that *L. monocytogenes* require synthesizing cobalamin under this stress condition. It has been reported that in either an aerobic or anaerobic condition, the cobalamin genes in *L. monocytogenes* may increase their relative expression in response to stress [27, 44]. Moreover, *eutV* expression increased in response to copper at low temperature with a similar pattern to what was observed for genes related to cobalamin synthesis. This result concurs with the role of cobalamin as a cofactor for the riboswitch Rli55, which controls the expression of the *eut* genes [45].

Recent reports indicate that the deletion of *cobK,* a gene that encodes a predicted precorrin-6A reductase, in *Mycobacterium smegmatis* affected cobalamin synthesis, suggesting that mutating one gene in the pathway affects cobalamin production [46]. Mutation of the gene *cbiP* (Δ*cbiP*) in *Halobacterium* reduced the archaeal growth in a medium deficient in corrinoids; however, when the medium was supplemented with cobyric acid, its growth ability was reestablished through reinstating the salvage pathway in the mutant strain [47].

CbiP is a synthase that catalyzes the synthesis of adenosyl-cobyric acid, a step that occurs almost at the end of the cobalamin biosynthesis pathway [48]. In this study, the gene *cbiP* of *L. monocytogenes* stressed by low temperature and copper showed high expression levels after 6 h at cold + copper. Deleting *cbiP* affected the growth of List-2-2 in a solid medium when bacteria were cultured at 8 °C + copper. The mutant reduced its growth with 1 mM copper, and it was inhibited at 3 mM copper compared to the wild-type strain. Supplementing the medium with cobalamin supported List2-2Δ*cbiP* growth as observed in the wild-type strain. This implies that *L. monocytogenes* may be using cobalamin from the medium to supply the needs to survive stress.

## Conclusions

These results suggest that cobalamin has a protective effect on the stress response of *L. monocytogenes* to cold and copper. Possible explanations may be that the antioxidant capacity of cobalamin helps to manage stress or that the lack of cobalamin would affect the ability of *L. monocytogenes* to metabolize other sources of energy (such as ethanolamine), affecting the survival of *L. monocytogenes*. Further studies to test these hypotheses and cobalamin concentrations in *L. monocytogenes* exposed to cold and copper will be helpful to elucidate the levels of vitamin required to protect against stress conditions.

## Methods

### Strains and culture conditions

*Listeria monocytogenes* strain List2-2 (NCBI biosample ID: SAMN19838404) was isolated from seafood and is part of our repository. The isolate was confirmed as *L. monocytogenes* by PCR [49]. The tolerance of List2-2 to low temperature and copper had been previously studied [35–37]. For assays at low temperature, List2-2 was adapted as follows: the first day, a single colony was inoculated into Trypticase Soy Broth (BBL, Becton Dickinson, United States) containing 0.6% yeast extract (Oxoid, Basingstoke, United Kingdom); TSBYe and cultured at 37 °C overnight at 160 rpm. The next day, cold, fresh TSBYe broth was inoculated with *L. monocytogenes* and adjusted to an optical density of 0.05 at 600 nm (OD_600nm_). The culture was grown at 8 °C (160 rpm) for 72 h at 160 rpm to adapt it to low temperatures. For assays at 37 °C, a single List2-2 colony was incubated in TSBYe at 37 °C (160 rpm) overnight. Microbiological assays were run in a biosafety level II-approved laboratory only accessible to trained individuals working with human foodborne pathogens.

### Identification of genes involved in the cobalamin pathway

We selected the genes that encode for proteins involved in the cobalamin pathway using the information for *L. monocytogenes* EGD-e strain (ID: AL591824) reported in the KEGG PATHWAY database (Kyoto Encyclopedia of Genes and Genomes). Then we searched for these genes in the genome of *L. monocytogenes* List2-2 strain by alignment with the BLAST tool (http://www.ncbi.nlm.nih.gov/BLAST).

### Gene expression assays

Three genes–lmo1201 (*cysG)*, lmo1208 (*cbiP*), and lmo1192 (*cbiB*)—involved in cobalamin biosynthesis were selected considering their position in the cobalamin biosynthesis gene cluster: the beginning, middle, and end. These genes encode proteins involved in the first and last stage of cobalamin biosynthesis, stages that operate under aerobic conditions (Additional file 1: Fig. S1). Moreover, the candidate genes were selected based on previous studies that have shown that the deletion of these genes affect cobalamin biosynthesis in other bacterial species [47, 50, 51]. We also evaluated the transcriptional response of *eutV,* a transcriptional regulator of the ethanolamine operon. *csoR* (lmo1854), a gene that encodes a transcription factor that regulates Cu homeostasis in *L. monocytogenes*, was used as a control to sense for copper exposure [36].

The expression level of these genes was evaluated in response to cold and copper. For this, a fresh TSBYe medium was inoculated with List2-2 adapted to low temperature and incubated at 8 °C/160 rpm to reach an early-log phase (OD_600nm_: 0.4). Then the medium was supplemented with a sub-inhibitory concentration of copper (CuSO_4_ × 5H_2_O) 0.5 mM and incubated at 8 °C/160 rpm under aerobic conditions. This copper concentration was used because it is non-lethal for *L. monocytogenes* but significantly increases the copper cellular content [36]. Samples were taken at 0 (before adding copper; the control condition), 1, 6, and 24 h for RNA extraction. RNA was extracted using the NucleoZOL reagent (Macherey–Nagel, Düren, Germany) according to the manufacturer's recommendations. Complementary DNA (cDNA) synthesis was carried out from 1 µg of RNA with the reverse transcriptase enzyme MMLV-RT (Moloney Murine Leukemia Virus Reverse Transcriptase; Promega, Madison, WI, USA). Primers were designed with the Primer-BLAST tool using the genome of *L. monocytogenes* List2-2 as a template (Additional file 2: Table S1). SYBR Green Agilent Master Mix enzyme (Agilent Technologies, Santa Clara, CA, USA) was used for qPCR amplification according to the manufacturer’s instructions. The qPCR conditions were an initial denaturation at 95 °C for 10 min, followed by 40 cycles of denaturation (95 °C for 30 s), annealing (60 °C for 60 s), and extension (72 °C for 30 s). Real-time PCR (qPCR) reactions were performed in the Agilent AriaMx Real-Time PCR system in a final reaction volume of 10 μl.

The relative abundance level of each evaluated transcript was compared at time zero to the other times tested and was calculated using the 2^−(ΔΔCt)^ method proposed by Livak and Schmittgen [52]. The 16S rRNA gene (*lmor04*) was used as housekeeping [36, 53]. The qPCR reaction for each gene was performed in three biological replicates with two technical replicates each.

### Generation of Δ*cbiP* mutant strain

*cbiP* was one of the genes that showed the highest expression levels in response to the stress condition in the study (8 °C + copper). It has been observed that its deletion affects the synthesis of cobalamin in other microorganisms [47]. Therefore, we evaluated the effect of the absence of a gene that encodes for CbiP in the tolerance to low temperature + copper in *L. monocytogenes* List2-2. A *cbiP* mutant was created with homologous recombination using a previously described protocol [54]. The vector pKSV7 was kindly provided by Dr. Rychli from the University of Veterinary Medicine, Vienna. The recombinant fragment (FP) was designed by SOE-PCR using primers detailed in Additional file 2: Table S1. Briefly, the fragment was inserted into the vector pKSV7 (pKSV7-FP). Electrocompetent *L. monocytogenes* List2-2 cells were transformed by adding 3 µg of the vector pKSV7-FP using the following electroporation parameters: 2.5 kV, 200 Ohms, 25 µF in a BioRad Gene Pulser X Cell electroporator (Biorad, Hercules, CA, USA). Multiple passages of transformed cells were performed at 40 °C in Trypticase Soy Broth (TSBYe) supplemented with chloramphenicol (10 μg/mL) to induce vector insertion. Vector excision was induced by cell passages in BHI broth at 30 °C without antibiotics. Possible mutants were first screened by PCR (primers cbiP_L_F-LV and cbiP_R_R-LV, Table 1), then the junction area was sequenced for confirmation.

### Mutant strain evaluation: tolerance to low temperature + copper

Overnight cultures of wild-type and List2-2Δ*cbiP* were diluted ten-fold to analyze their growth in agar. A volume of 10 μL from the 10^–4^ to the 10^–6^ dilutions were inoculated on TSAYe agar plates supplemented with 1 and 3 mM CuSO_4_ × 5H_2_O. Copper-free TSAYe agar was used as a control. A 10 μL volume from the 10^–4^ to 10^–7^ dilutions was also inoculated in plates supplemented with 5 nM of cyanocobalamin (Merck KGaA, Darmstadt, Germany) to test the protective role of cyanocobalamin against the cold + copper stress on *L. monocytogenes.* All plates were incubated at 8 °C, and the growth was monitored every day for 7 days. Each assay was performed in triplicate.

## Statistical analysis

Statistical analysis was conducted in R project 4.0.2 [55]. Relative expression data were described as mean ± standard deviation and all comparisons were performed between times (1, 6, and 24 h). Non-parametric Kruskal–Wallis and post hoc Dunn tests were used to analyze the statistical significance between experimental groups. *P-values* < 0.05 were considered statistically significant.

## Supplementary Information

**Additional file 1: Figure S1.** Cobalamin biosynthesis pathway in *L. monocytogenes*. **(A)** Stages of the cobalamin biosynthesis pathway in *L. monocytogenes* List2-2. Cobalamin biosynthesis can be divided into three stages following the classification of Scott & Roessner, 2002 [24]. Stage 1: The transformation of Uroporphyrinogen III to Precorrin-2, which is similar for both the anaerobic and the aerobic, and is carried out by the CysG / CobA proteins; Stage 2: This is different for the anaerobic route (*L. monocytogenes*), where the CysG protein (bifunctional protein) inserts the cobalt ion in the precorrin-2 molecule independently of oxygen. For the aerobic pathway (right side) the cobalt ion is inserted into the molecule by the CobNST complex, and Stage 3: Both pathways (aerobic and anaerobic) converge in cob(II)yrinate a, c diamide which is finally transformed into cobalamin. **(B)** Arrangement of the cluster of genes involved in cobalamin biosynthesis in *L. monocytogenes* List2-2 genome. ***(i)*** Twenty genes encoded in a cluster of 16870 bp; ***(ii)*** Two genes of the cobalamin biosynthetic pathway encoded in the propanediol operon.

**Additional file 2: Table S1.** Primers used in this study.

## Data Availability

Not applicable

## Abbreviations

*L. monocytogenes*: *Listeria monocytogenes*
List2-2: *L. monocytogenes* Strain
*cbiP*: A cobyric acid synthase encoding gene
*cbiB*: A cobalamin biosynthesis encoding gene
*cysG*: A uroporphyrin-III C-methyltransferase encoding gene
*eutV*: A transcriptional regulator encoding gene
Δ*cbiP*: List2-2 mutant for *cbiP* gene
PCR: Polymerase chain reaction
qPCR: Quantitative real-time PCR
TSBYe: Trypticase soy broth and yeast extract
OD_600nm_: Optical density at 600 nm
