# Supplementary figures and images for: Cobalamin cbiP mutant shows decreased tolerance to low temperature and copper stress in Listeria monocytogenes
Source: Biol Res. 2022 Mar 2;55:9. doi: 10.1186/s40659-022-00376-4 (PMC8889760; doi:10.1186/s40659-022-00376-4)

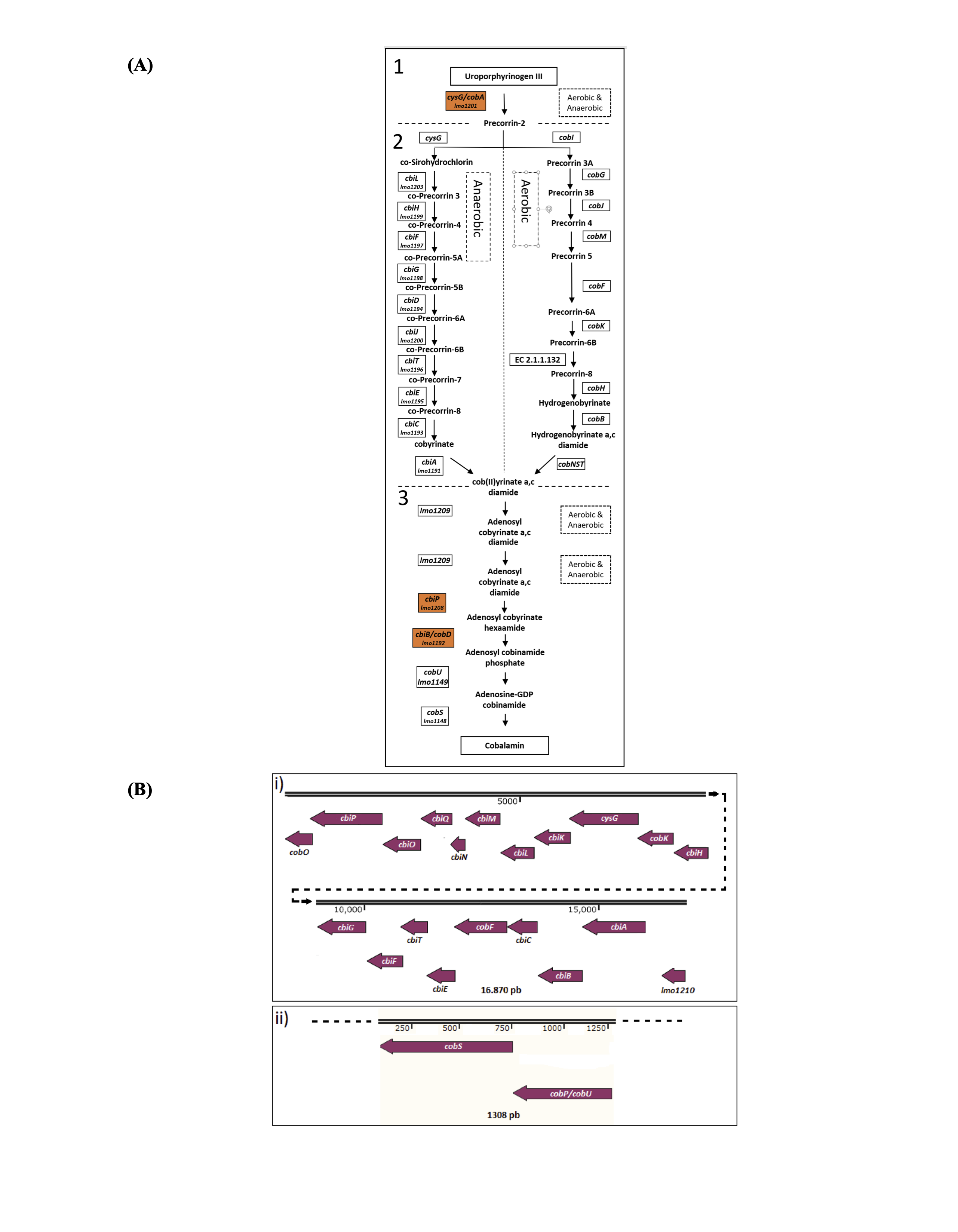

Supplement: Supplementary file 1 — Additional file 1: Figure S1. Cobalamin biosynthesis pathway in L. monocytogenes. (A) Stages of the cobalamin biosynthesis pathway in L. monocytogenes List2-2. Cobalamin biosynthesis can be divided into three stages following the classification of Scott & Roessner, 2002 [24]. Stage 1: The transformation of Uroporphyrinogen III to Precorrin-2, which is similar for both the anaerobic and the aerobic, and is carried out by the CysG / CobA proteins; Stage 2: This is different for the anaerobic route (L. monocytogenes), where the CysG protein (bifunctional protein) inserts the cobalt ion in the precorrin-2 molecule independently of oxygen. For the aerobic pathway (right side) the cobalt ion is inserted into the molecule by the CobNST complex, and Stage 3: Both pathways (aerobic and anaerobic) converge in cob(II)yrinate a, c diamide which is finally transformed into cobalamin. (B) Arrangement of the cluster of genes involved in cobalamin biosynthesis in L. monocytogenes List2-2 genome. (i) Twenty genes encoded in a cluster of 16870 bp; (ii) Two genes of the cobalamin biosynthetic pathway encoded in the propanediol operon. [file 40659_2022_376_MOESM1_ESM.png]
