# Supplementary material for: Cobalamin cbiP mutant shows decreased tolerance to low temperature and copper stress in Listeria monocytogenes
Source: Biol Res. 2022 Mar 2;55:9. doi: 10.1186/s40659-022-00376-4 (PMC8889760; doi:10.1186/s40659-022-00376-4)
Supplement: Supplementary file 2 — Additional file 2: Table S1. Primers used in this study. [file 40659_2022_376_MOESM2_ESM.docx]

**Supplementary Table 1** Primers used in this study

| Name | Sequence (5’-3') | Length (bp) |
| --- | --- | --- |
| ***qPCR primers*** | | |
| ***cysG***  *cysG*_QLV_F  *cysG*_QLV_R | AATTCCGTGCTCCGTCTTTT  TCTCTTAAAAATAGCCATCTGACG | 107 |
| ***cbiB***  *cbiB*_QLV_F  *cbiB*_QLV_R | CGAAGTACTCATGCGTTCCA  GCGAGTAGGTCTCCGTGATA | 113 |
| ***cbiP***  *cbiP*_QLV_F  *cbiP*_QLV_R | TTTGCGCGTTAGTAGTTCGT  GGCGCTATCTCTGTCAACAA | 105 |
| ***eutV***  *eutV*_QLV_F  *eutV*_QLV_R | CCAGCAAGCCCTTCTGAAAT  GCGACAGATGGTTTTGAAGC | 131 |
| ***csoR***  *csoR*_FW  *csoR*_RV | AATCCGCGGTATTGCTCAAA  TTGCTGCATCTACAACACAGT | 146 |
| ***Primers for SOE-PCR mutagenesis*** | | |
| *cbiP*_L_F-LV*  *cbiP*_L_R-LV | AGCGGAATTCCACTTCTTCGTTACTTGAAGC  ACAAGAATTATCGGGGGTAG | 405 |
| *cbiP*_R_F-LV  *cbiP*_R_R-LV** | CCCGATAATTCTTGTTTAATTAGTCACCTTCCCCGTA  TCGGGATCCGTGCTTAAGATTGTTGGTGC | 447 |

Star indicate restriction sites: * *EcoR*I; ** *BamH*I.

Underline nucleotides indicates the specific enzyme restriction site.
